# Exosome-encapsulated ncRNAs: Emerging yin and yang of tumor hallmarks

**DOI:** 10.3389/fgene.2022.1022734

**Published:** 2022-10-20

**Authors:** Nazoora Khan, Mohd Saad Umar, Mohamed Haq, Talha Rauf, Swaleha Zubair, Mohammad Owais

**Affiliations:** ^1^ Interdisciplinary Biotechnology Unit, Aligarh Muslim University, Aligarh, India; ^2^ University of Houston, Houston, TX, United States; ^3^ Department of Computer Science, Faculty of Science, Aligarh Muslim University, Aligarh, India

**Keywords:** exosome, non-coding RNA, tumor-hallmarks, tumor promoter, tumor suppressor

## Abstract

Tumorigenesis is a multifaceted process, where multiple physiological traits serving as cancer’s distinctive characteristics are acquired. “Hallmarks of cancer” is a set of cognitive abilities acquired by human cells that are pivotal to their tumor-forming potential. With limited or no protein-coding ability, non-coding RNAs (ncRNAs) interact with their target molecules and yield significant regulatory effects on several cell cycle processes. They play a “yin” and “yang” role, thereby functioning both as oncogenic and tumor suppressor and considered important in the management of various types of cancer entities. ncRNAs serve as important post-transcriptional and translational regulators of not only unrestricted expansion and metastasis of tumor cells but also of various biological processes, such as genomic mutation, DNA damage, immune escape, and metabolic disorder. Dynamical attributes such as increased proliferative signaling, migration, invasion, and epithelial–mesenchymal transition are considered to be significant determinants of tumor malignancy, metastatic dissemination, and therapeutic resistance. Furthermore, these biological attributes engage tumor cells with immune cells within the tumor microenvironment to promote tumor formation. We elaborate the interaction of ncRNAs with various factors in order to regulate cancer intra/intercellular signaling in a specific tumor microenvironment, which facilitates the cancer cells in acquiring malignant hallmarks. Exosomes represent a means of intercellular communication and participate in the maintenance of the tumor hallmarks, adding depth to the intricate, multifactorial character of malignant neoplasia. To summarize, ncRNAs have a profound impact on tumors, affecting their microcirculation, invasiveness, altered metabolism, microenvironment, and the capacity to modify the host immunological environment. Though the significance of ncRNAs in crosstalk between the tumor and its microenvironment is being extensively explored, we intend to review the hallmarks in the light of exosome-derived non-coding RNAs and their impact on the tumor microenvironment.

## Introduction

A continuous physiochemical balance between various parts of the body is sought after by all living organisms. The body maintains homeostasis by the release of a variety of vesicles, including apoptotic bodies, shed microvilli, microparticles, ectosomes, and exosomes, comprising a wide variety of components (Mathieu et al., 2019). Exosomes are produced within multi-vesicular bodies (MVBs) or multi-vesicular endosomes and are secreted upon their fusion with the plasma membrane (McKelvey et al., 2015). The majority of “normal cell” types, such as mast cells, dendritic cells, reticulocytes, epithelial cells, B-cells, trophoblastic cells, and neural cells and a variety of malignant cell types produce exosomes (40–150 nm diameters) (Kalluri and LeBleu, 2020). Exosomes were initially considered conduits for evacuation of waste products from cell, but recent scientific investigations consistently show their involvement in a myriad of critical physiologic processes (Rajagopal and Harikumar, 2018). Upon budding off from the cell, the exosomal contents are guarded from the detrimental extracellular conditions by their sturdy lipid membrane (Steinbichler et al., 2017). Exosomes as nomadic vesicles alter the function and phenotype of the recipient cell *via* trafficking to distant and proximal sites and can target recipient cells owing to the molecules on their surface (Brinton et al., 2015). Exosomes can be internalized by cells through endocytosis and/or phagocytosis once they are in close proximity to a cell, in addition to triggering signaling through receptor–ligand interaction. Additionally, upon fusion of the exosome with the recipient’s membrane, their payload is released into the cytosol of the recipient cell (Horibe et al., 2018).

Exosomes can either be a part of tumor cell secretions or stromal cell secretions, depending on their origination in the tumor microenvironment (Penfornis et al., 2016). They are erratically released in large quantities by cancer cells, which serve as a reflection of the stromal cells’ phenotypic condition. The content of exosomes changes dynamically as the tumor progresses (Tzaferi et al., 2021). Within the tumor microenvironment, the secretion of exosomes by tumors promotes crosstalk or communication between tumor cells and cells like fibroblasts, endothelial cells, mesenchymal stromal cells, cancer stem cells, and immune cells (López de Andrés et al., 2020). Exosome internalization by recipient cells appears to be a cell-type-specific process, and the degree of internalization is likely dependent on the recipient cell’s phagocytic capacity (Milane et al., 2015). Exosomes can trigger target cells in the following ways—1) direct stimulation mediated by surface-expressed ligands, 2) through transfer of receptors from tumor cells to target cells, 3) through horizontal transfer of genetic material to target cells, and 4) through direct stimulation mediated by receptor-mediated endocytosis (Teng and Fussenegger, 2021). Exosomal movement between cells and the tumor microenvironment may exert a profound biological effect, accelerating the development of tumors and metastatic spread *via* the release of growth factors, cytokines, proteins, lipids, and non-coding RNAs (ncRNAs) (Steinbichler et al., 2017).

**FIGURE 1:**
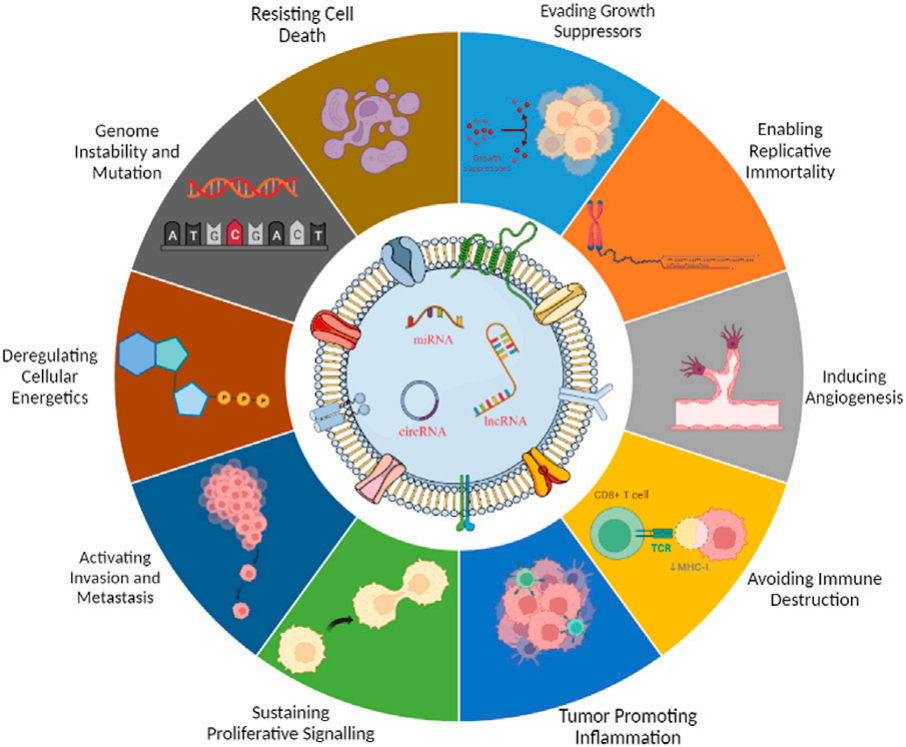
Exosomal ncRNAs are involved in the hallmarks of cancer. Perturbed ncRNAs may act as oncogenes by promoting hallmarks of cancer or as tumor suppressors by constraining them.

Hanahan and Weinberg codified the concept that normal cells transform progressively to the neoplastic stage via acquiring particular hallmarks eventually (Hanahan and Weinberg, 2011). Recent reports suggest about the eight different hallmarks acquired during tumorigenesis, namely, proliferation (evading), growth suppression, viability, immortality, angiogenesis, motility, energy metabolism, and immune evasion (Gutschner and Diederichs, 2012). The anomalous state of neoplasia, which offers a mechanism for cancer cells and tumors to adopt these functional properties, has led to the addition of a new concept, portrayed as “enabling characteristics.” In this way, along with the aforementioned eight hallmarks, genomic instability and tumor-promoting inflammation considered enabling characteristics, reflected upon molecular and cellular pathways through which the hallmarks are acquired (Hanahan, 2022). A deeper insight of cancer propagation and acquirement hallmarks suggests the role of cancer cell-derived exosome-based payloads (Meehan and Vella, 2016). For the scope of this review, we have assessed the potential of non-coding RNA-loaded exosomes in modulation of cancer hallmarks (Figure 1).

## Exosomal biogenesis and composition

Within multi-vesicular bodies, inward budding of the late endosomal membrane produces intraluminal vesicles (exosomes) with varied payloads, which are emancipated into the extracellular environment upon fusion with the cellular membrane (Théry et al., 2002).

**FIGURE 2:**
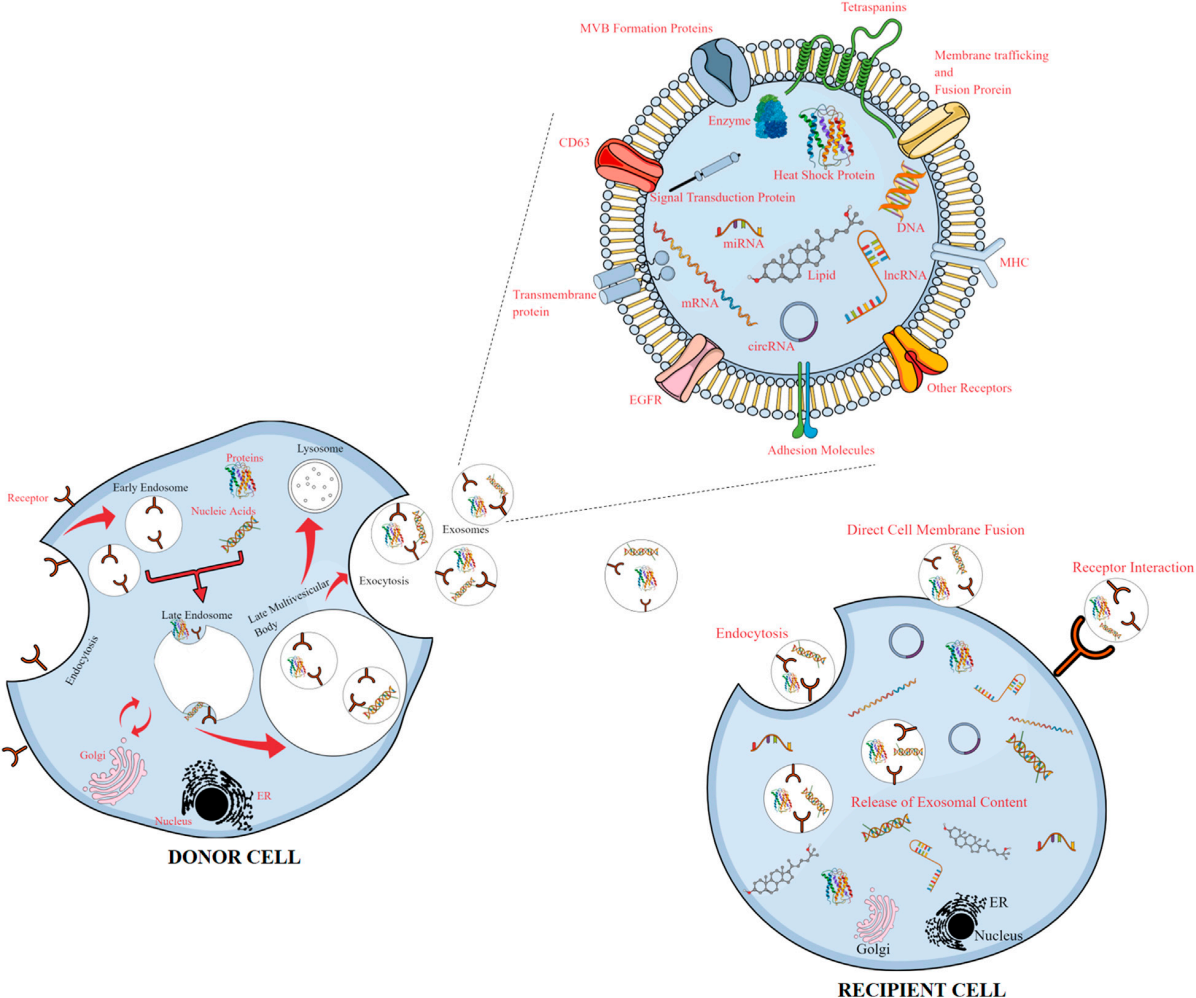
Release of exosomes from donor cells and uptake by recipient cells, with an enlarged view of the exosomal content. Exosomes released from donor cells carry cargos of proteins, lipids, and genetic materials and can be taken up by recipient cells, reprogramming the recipient cells upon transfer of their bioactive compounds.

Exosomal biogenesis primarily entails three phases: initially, invagination of the plasma membrane forms an early endosome, enclosing endocytic payloads like soluble and cell surface proteins (Barile and Vassalli, 2017) (Figure 2). To foster the development of endosomes, the endosomal sorting complex required for transport (ESCRT) mechanism is considered a critical circuitry for the formation of MVEs and release of exosomes (Baietti et al., 2012). The ESCRT comprises four complexes, namely, ESCRT-0, I, II, and III. While ESCRT-I and ESCRT-II are in control of squishing the membrane to generate a stable membrane neck, ESCRT-0 assembles ubiquitin cargo proteins into lipid domains. Vesicular neck segmentation and ESCRT-III severance and salvaging are triggered by association of the VPS4 complex into ESCRT-III. An activated ALIX protein may recruit ESCRT-III proteins to endosomes, while TSG101 has been associated to exosome release (Vella et al., 2008). Numerous publications have also established that lipids and related proteins are used during exosome synthesis and cargo loading in an ESCRT-independent mechanism (Hessvik and Llorente, 2018).

The process of RNA loading inside exosomes is believed to be lipid-dependent and necessitates the presence of a set of independent lipids and cargo domains. Particular sequences of nucleotides, like those found in lipid rafts, hydrophobic modifications, or sphingosine, have an increased affinity for the phospholipid bilayer (Wei et al., 2021). The binding of proteins or other molecules to lipid rafts, which are rich in cholesterol, sphingolipids, and glycosylphosphatidylinositol-anchored proteins, may increase their secretion through exosomes (de Gassart et al., 2003). Intraluminal vesicle (ILV) production also takes place due to the presence of ceramide, lysophosphatidic, and glycosphingolipid molecules on the limiting membrane. Stimulation of S1P receptors promotes the conciliation of tetraspanin sorting into ILVs (Yue et al., 2020; He et al., 2022). Ceramide kinase and ceramidase could metabolize ceramide into sphingosine and sphingosine-1-phosphate (S1P). Tetraspanin-enriched micro-domains, which are membrane micro-domains, abundant in transmembrane and cytoplasmic signal proteins, are organized by the tetraspanin superfamily, comprising membrane proteins with transmembrane domains (Ogretmen, 2018). Lack of ESCRT machinery may cause the sorting of cargo into ILVs and variation in the amount and size of ILVs (Raiborg and Stenmark, 2009), thus implying that exosome biogenesis may involve both ESCRT-dependent and -independent processes in a cohesive way.

Exosomes are usually characterized by electron microscopy (SEM/TEM/CryoEM), atomic force microscopy (AFM), dynamic light scattering (DLS), nanoparticle tracking analysis technology (NTA), fluorescence correlation spectroscopy (FCS), resistive pulse sensing, western-blot, enzyme-linked immunosorbent analysis (ELISA), and flow cytometry. The vesicular constituent of exosomes includes proteins, DNA (mtDNA, ssDNA, and dsDNA), and RNA (mRNA, miRNA, lncRNA, and circRNA) of the host origin and even genetic material of malignant cells and pathogens. Of these, the encapsulated proteins can be classified into two broad categories, namely, specific and non-specific proteins (Patil and Rhee., 2019). The majority of non-specific proteins (like, annexins, flotillins, MHC I and II, and heat shock proteins 70/90) arise from parental cell cytoplasmic and conserved membrane proteins essential for the formation and functioning of exosomes. Specific proteins have been found to be correlated with their origin; for example, exosomes derived from the T lymphocyte possess granular enzymes and perforin proteins on their surface (Mashouri et al., 2019). Notably, exosomes possess a wide array of RNAs that are responsible for execution of various biological functions. Among these, the non-coding RNAs, once regarded as junk, regulate the gene expression of the critical biological processes at the genomic and transcriptomic levels (Yue et al., 2020). The ESCRT proteins recruit several non-coding RNAs to be encapsulated into the exosomes. With the advent of NGS technologies, the exosomes derived from different biological fluids like saliva, CSF, plasma cells, serum, and urine were found to possess snRNAs, circRNAs, snoRNAs, piRNAs, miRNAs, lncRNAs, transfer RNAs, and ribosomal RNAs (Cheng J. et al., 2020). In contrast to the free form of ncRNAs, exosomes safeguard the encapsulated ncRNAs from enzymatic degradation, facilitating the execution of their biological functions. Some ncRNAs integrate functionally into a variety of important cell growth pathways. Their context-dependent deregulation in cancer suggests that ncRNAs play both oncogenic and tumor suppressive roles. (Fan et al., 2018) (as shown in Table 1).

**TABLE 1:** Tumor-promoting and tumor-suppressing roles of exosomal ncRNAs in hallmarks of cancer.

Cancer hallmark	ncRNA	Cancer	Target	Role		Reference
				Tumor promoter	Tumor suppressor	
Evading growth suppressors and sustaining proliferative signaling	miR-1246	Breast	CCNG2	✓		Li et al. (2017)
	miR-96	Lung	LMO7	✓		Wu et al. (2017)
	hsa-miR199a-3p	Neuroblastoma	NEDD4	✓		Ma et al. (2019)
	miR-143-3p	Lung	ITM2B	✓		Zhou et al. (2020)
	miR-9-3p	Bladder	ESM1		✓	Cai et al. (2019)
	miR-133b	Bladder	DUSP1		✓	Cai et al. (2020)
	miR-144	NSCLC	CCNE1		✓	Liang et al. (2020)
	miR-744	HCC	PAX2		✓	Wang et al. (2019)
	miR-375-3p	Bladder	FZD8		✓	Li et al. (2020)
	miR-204-5p	Breast, glioma, lung, and gastric	RAB22A and Bcl2		✓	Yao et al. (2020)
	lncRNA HOTAIR	Lung	miR-203	✓		Zhang et al. (2020)
	lncRNA UFC1	NSCLC	EZH2/PTEN miR-124	✓		Zang et al. (2020)
	lncRNA ZFAS1	Gastric	miR-1236	✓		Pan et al. (2017)
	lncRNA FAL1	HCC	miR-182-5p/FOXO3	✓		Li et al. (2018)
	lncRNA LBX1-AS1	OSCC	miR-106a-5p		✓	Ai et al. (2021)
	lncRNA HAND2-AS1	Breast	miR-17		✓	Xing et al. (2021)
	lncRNA PTENP1	Bladder			✓	Zheng et al. (2018)
	circMAN2B2	HCC	miR-217	✓		Fu et al. (2020)
	circARHGAP10	NSCLC	miR-638	✓		Fang et al. (2022)
	circNRIP1	Gastric	miR-149-5p	✓		Zhang et al. (2019)
	circ-0051443	HCC	miR-331-3p		✓	Chen et al. (2020)
	hsa_circ_0072309	Gastric	PPARγ/PTEN		✓	Guo et al. (2022)
Resisting cell death	miR-205	Ovarian	VEGFA	✓		Wang et al. (2019)
	miR-4535	Melanoma	ATG13	✓		Liu et al. (2022)
	miR-224-5p	Breast	HOXA5	✓		Wang et al. (2021)
	miR-25	HCC	SIK1	✓		Fu et al., (2022)
	miR-181d-5p	Breast	CDX2 and HOXA5	✓		Wang et al. (2020)
	miR-148b-3p	Bladder	Wnt/β-catenin	✓		Shan et al. (2021)
	miR-1910-3p	Breast	MTMR3		✓	Wang et al. (2020)
	miR-451a	HCC	LPIN1		✓	Zhao et al. (2019)
	lncRNA CEBPA-AS1	Gastric	CEBPA/BCL miR-15a/16 and BCL-2	✓		Piao et al. (2020)
	lncRNA LINC00461	Myeloma	miR-122-5p/XIAP	✓		Deng et al. (2019)
	lncRNA SBF2-AS1	Pancreatic	miR-580-3p/WEE1	✓		Yin et al. (2020)
	lncRNA LINC00470	GBM	YBOX3/P21	✓		Ma et al. (2021)
	lncRNA SNHG9	PTC	ULK1 miR-153/ATG5		✓	Wen et al. (2021)
	lncRNA H19	Bladder			✓	Guo et al. (2022)
	lncRNA OIP5-AS1	Osteosarcoma			✓	Li et al. (2021)
	circRNA_400068	Renal	miR-210-5p/SOCS1	✓		Xiao and Shi., 2020
	circ-PVT1	Gastric	miR-30a-5p/YAP1	✓		Yao et al. (2021)
	circ-UBE2Q2	Gastric	STAT3	✓		Yang et al. (2021)
	circRELL1	Gastric	EPHB3/miR-637	✓		Sang et al. (2022)
Enabling replicative immortality	miR-185	Fibro sarcoma	POT1	✓		Li et al. (2020)
	miR-22	Cervical	MYCBP		✓	Konishi et al. (2020)
	lncRNA TERRA	Colon	Telomerase		✓	Wang et al. (2015)
	circWHSC1	Ovarian	MUC1 and hTERT	✓		Zong et al. (2019)
Inducing angiogenesis	miR-141	Lung	GAX	✓		Wang et al. (2021)
	miR-23a	Gastric	PTEN	✓		Du et al. (2020)
	miR-619-5p	NSCLC	RCAN1	✓		Kim et al. (2020)
	miR-1290	HCC	SMEK1	✓		Wang et al. (2021)
	miR-210	HCC	SMAD4 and STAT6	✓		Lin et al. (2018)
	miR-9	NPC	MDK		✓	Lu et al. (2018)
	lncRNA RAMP2-AS1	Chondrosarcoma	miR-2355-5p/VEGFR2	✓		Cheng et al. (2020)
	lncRNA UCA1	Pancreatic	miR-96-5p/AMOTL2/ERK1/2	✓		Guo et al. (2020)
	lncRNA FAM225A	ESCC	miR-206/NETO2 and FOXP1	✓		Zhang et al. (2020)
	circRNA-100338	HCC	NOVA2 miR-29a	✓		Huang et al. (2020)
	circRNA-29	Gastric		✓		Li et al. (2021)
Activating invasion and metastasis	miR-208a	Osteosarcoma	PDCD4	✓		Qin et al. (2020)
	miR-1246	OSCC	DENND2D	✓		Sakha et al. (2016)
	miR-92a-3p	HCC	PTEN	✓		Yang et al. (2020)
	miR-3940-5p	CRC	ITGA6		✓	Li et al. (2021)
	miR-3607-3p	Pancreatic	IL-26		✓	Sun et al. (2019)
	lncRNA HOXD-AS1	Prostate	miR-361-5p/FOXM1	✓		Jiang et al. (2021)
	lncRNA PCGEM1	Gastric	SNAI1 miR-326/FSCN1	✓		Piao et al. (2021)
	lncRNA LINC01711	ESCC		✓		Xu et al. (2021)
	circ-0004277	HCC	ZO-1 miR-133a/GEF-H1/RhoA	✓		Zhu et al. (2021)
	circ-133	CRC	miR-653-5p/PAX6	✓		Yang et al. (2020)
	circ007293	PTC	miR-338/MACC1/MET/AKT or ERK	✓		Lin et al. (2021)
	circ-PDE8A	PDAC		✓		Li et al. (2018)
Reprogramming of energy metabolism	miR-105	Breast	MYC	✓		Yan et al. (2018)
	miR-155 and miR-210	Melanoma	OXPHOS	✓		Shu et al. (2018)
	miR-21-5p	Ovarian	PDHA1	✓		Zhuang et al. (2021)
	lncRNA SNHG3	Breast	miR-330/PKM		✓	Li et al. (2020)
	ciRS-122	Colorectal	miR-122/PKM2	✓		Wang et al. (2020)
	circ_0094343	Colorectal	TRIM67		✓	Li and Li., 2022
Evading immune destruction and tumor-promoting inflammation	miR-1468-5p	Cervical	HMBOX1-SOCS1	✓		Zhou et al. (2021)
	miR-1290	Gastric	Grhl2/ZEB1/PD-L1	✓		Liang et al. (2021)
	miR-222	CRC	ATF3	✓		Li et al. (2021)
	miR-675-3p	Gastric	CXXC4	✓		Li et al. (2020)
	miR-21	Glioma	PEG3	✓		Yang et al. (2020)
	miR-15a	CRC	KDM4B and HOXC4		✓	Liu et al. (2021)
	miR-186	Neuroblastoma	TGFβ1		✓	Neviani et al. (2019)
	lncRNA SNHG16	Breast	miR-16–5p	✓		Ni et al., (2020)
	lncRNA SNHG10	CRC	INHBC	✓		Huang et al. (2021)
	lncRNA KCNQ1OT1	CRC	PD-L1	✓		Xian et al. (2021)
	lncRNA TUC339	HCC	CXCR miR-34/mir-449-5p	✓		Li et al. (2018)
	lncRNA ARSR	RCC		✓		Zhang et al. (2022)
	circUHRF1	HCC	TIM-3/miR-449c-5p miR-934/SHP2	✓		Zhang et al. (2020)
	circUSP7	NSCLC	miR-324-5p/TGFBR1/Smad3	✓		Chen et al. (2021)
	circGSE1	HCC	S100A11 miR141-3p/GLS1	✓		Huang et al. (2022)
	circ_6790	PDAC			✓	Gao et al. (2022)
	circTRPS1	Breast			✓	Yang et al. (2022)

## ncRNA Biogenesis

ncRNAs are a class of functional regulatory RNA molecules lacking the ability to code for proteins (Ferreira and Esteller, 2018). They are classified according to length (small: 18–200 nt; long: more than 200 nt) or by function (housekeeping ncRNAs, including rRNAs and tRNAs) and regulatory transcripts like miRNA, lncRNA, and circRNA. Substantial mounting evidences suggest that non-coding RNAs, considered ‘dark matter of the genome,’ control several critical biological processes through careful manipulation of key biochemical pathways (Diederichs et al., 2016).

miRNA biogenesis initiates with transcription of genes into large primary transcripts mediated by RNA polymerase II/III during or post-transcription. The discovered miRNAs until now are categorized broadly into three types, namely, intragenic, intergenic, and exonic (Liu et al., 2019). The regulation of intra and exo-genic miRNA is dependent on the host promoter and is processed from introns and exons, while for intragenic miRNAs, the transcription process is independent of the host and regulated by their own promoters. Canonically, miRNAs are transcribed by introns of coding or non-coding transcripts, and few miRNAs are transcribed by exonic regions. Initially, transcription of miRNA genes leads to generation of 5′ capped and 3′ polyadenylated pri-miRNA transcripts. Subsequent processing of pri-miRNA is orchestrated by the microprocessor complex [comprising DiGeorge syndrome critical region 8 (DGCR8—an RNA binding protein) and DROSHA (a ribonuclease III enzyme] inside the nucleus. DGCR8 mediates recognition of GGAC and other specific motifs within the pri-miRNA, and DROSHA mediates the digestion of pri-miRNAs, consequently generating stem-loop-like structures termed as pre-miRNAs (O'Brien et al., 2018). The export of pre-miRNA from the nucleus to cytosol is mediated by the exportin5/RAN/GTP complex and is cleaved by the DICER/TRBP/PACT complex favoring the formation of an miRNA duplex. The miRNA duplex is then loaded into the RISC complex in order to unwind the duplex structure with the incorporation of argonaute protein. After unwinding of the duplex, the mature miRNA is incorporated into RNA-induced silencing complex and guides the complex toward target mRNA for gene silencing or translation repression (Rani and Sengar, 2022).

Non-canonically, miRNA biogenesis falls into two categories, namely, Drosha/DGCR8-independent and Dicer-independent process. Within these groups, different plausible combinations of the proteins, namely, Drosha, Dicer, Exportins, and Argonaute involved in canonical pathways are utilized for the transcription. In the Drosha/DGCR8-independent pathway, the miRNAs termed as mirtrons are generated *via* the splicing-dependent process, replacing the microprocessor step from the introns of host mRNA (Titov and Vorozheykin, 2018). Post splicing, the lariat is de-branched and refolds into a stem-loop-like structure, resembling a pre-miRNA. These are transported to the cytoplasm *via* exportin 5 without the cleavage by Drosha. In the Dicer-independent mechanism, Drosha processes miRNAs from endogenous short-hairpin RNA transcripts. Owing to the fact that these pre-miRNAs lack the requisite length to serve as dicer substrates, the maturation process within the cytoplasm requires the presence of AGO2. As a result, the subsequent loading of pre-miRNA into AGO2 and splicing of the 3p strand is facilitated. The maturation step is accomplished by the 3′–5′, shortening of the 5p strand (Stavast and Erkeland, 2019; Treiber et al., 2019).

Long non-coding RNAs (lncRNAs) are quintessential RNA-like molecules with 3′ poly(A) tail and 5’methyl cytosine capping that are transcribed by RNA Pol II (Quinn and Chang, 2016). They are classified according to their wide range of features. Based on chromosomal position—sense, antisense, bidirectional, intronic, and intergenic; based on their function—signals, decoys, guides, and scaffolds, and based on their subcellular localization, lncRNAs are categorized into nuclear, cytoplasmic, and mitochondrial lncRNAs (Wu et al., 2017; Dahariya et al., 2019).

The biosynthesis of lncRNAs is akin to that of mRNA, along with some mechanical differences. The lncRNA transcriptional process includes 5’-capping, 3’-polyadenylation, RNA-editing processes, regular and alternative splicing mechanisms, and patterns of transcriptional activation. It has been shown that the vast majority of lncRNAs adhere to the canonical structure, implying that they are all capped, polyadenylated, and spliced (Chen, 2016). Some non-canonical mechanisms may also play a role, such as the formation of circular structures, capping by snoRNA-protein (snoRNP) complexes, and cleavage by ribonuclease P (RNase P), which results in mature 3′ ends (Xing and Chen, 2018). The production of lncRNAs is controlled by a wide variety of epigenetic changes and a variety of different regulators.

circRNAs can stem from either the exons or the introns of a gene, which then leads to the production of distinct categories of circRNAs: exonic, intronic, and exon–intronic. Exonic circRNAs are produced following a process called pre-mRNA splicing. During this process, the 3′ splice donor is joined to the 5′ splice acceptor, which results in the development of an exonic circRNA (Lu, 2020). Under certain conditions, it will merely consist of a single exon, while in others, the beginnings of an upstream exon will be spliced onto the end of a downstream exon. Afterward, the interceding RNA is circularized, leading to the generation of circRNAs from multiple exons (Ragan et al., 2019). On the other hand, if the intron that is located between the exons is preserved, the circular transcript that results is called exon–intron circRNA. The last possibility is that intronic circRNAs are generated from intron lariats that are degradation-resistant by de-branching enzymes. Intronic circRNAs are distinguished from exonic circRNAs by the presence of a singular characteristic 2′–5′ linkage within their structure (Barrett et al., 2015). The generation of intronic circRNAs is dependent on the presence of GU-rich sequences in close proximity to the 5′ splice site and C-rich sequences in close proximity to the branch point in the gene. During back-splicing, the two segments will initially come together to form a circle. Subsequently, the exonic and intronic sequences found in the binding region will be removed by the spliceosome, and the trailing introns will be brought together to produce intronic circRNA (Qu et al., 2015).

## Exosomal ncRNAs in regulating cancer hallmarks

### Evading growth suppressors and sustaining proliferative signaling

Aberrant cell proliferation is the most crucial hallmark of cancer. Any abnormality in the cell cycle of the given cell population is the prominent cause of tumorigenesis (Fouad and Aanei, 2017). Mechanistically, cell cycle progression is regulated by both intracellular and extracellular signal molecules, in order to achieve the balance between cell proliferation and cell cycle arrest (Liu et al., 2021). The cells become cancerous when cell growth or division becomes uncontrolled.

#### miRNAs

miRNAs are often stable within exosomes because they are not degraded by RNAse enzymes. miRNAs transported by exosomes can influence tumor growth and participate in different processes of tumorigenesis and tumor development. Exosomal miR-1246 induces a tumor-promoting phenotype, positively correlated with enhanced cell proliferation by directly targeting CCNG2 expression *via* binding to its 3′UTR (Li et al., 2017). miR-96 is increased in lung tissues and serum exosomes isolated from lung cancer patients and is positively correlated with cancer risk, promoting its progression. LMO7 is the direct target of miR-96, whose overexpression reverses the promoting effect of miR-96 in lung cancer (Wu et al., 2017). Exosomal hsa-miR 199-3p has the ability to enhance the proliferative nature of cancer by downregulating the NEDD4 level in neuroblastoma, indicating that exosomal hsa-miR199a-3p might be associated in the future development of novel therapeutic strategies for neuroblastoma (Ma et al., 2019). Granulocytic myeloid-derived suppressor cells (G-MDSCs) profusely secrete exosomes in the lung cancer tissues, which promotes cell proliferation ensuing in cancer progression. G-MDSC-derived exosomes, loaded with miR-143-3p, targets the 3ʹ-untranslated region (UTR) of integral membrane protein 2B (ITM2B), and hence, overexpression of miR-143-3p induces cell proliferation by suppressing ITM2B transcription and activating the PI3K/Akt signaling pathway (Zhou et al., 2020).

Along with the oncogenic miRNAs, certain exosomal miRNAs have been found to exert tumor-suppressive effects. The potential regulatory role of miR-9-3p in bladder cancer has been deciphered, and miR-9-3p delivered from bone marrow-derived mesenchymal stem cell (BMSC)-secreted exosomes is found to exert antitumor effects by suppressing a tumor promoter gene ESM1 (Cai et al., 2019). The exosomal miR-133b targets DUSP1 and, thereby, inhibits bladder cancer (BC) proliferation (Cai et al., 2020). miR-144 derived from bone marrow mesenchymal stem cell (BMMSC) exosomes can decrease the levels of CCNE1 and CCNE2, hence repressing the proliferation of NSCLC (Liang et al., 2020). miR-744 has downregulated exosomal expression in hepatocellular carcinoma (HCC). Moreover, PAX2, an overexpressed gene, is directly targeted by miR-744 and downregulated miR-744, aids in the propagation of HCC cells. Specifically, the propagation of HCC cells got substantially suppressed upon treatment with miR-744-loaded exosomes (Wang et al., 2019). The miRNA profile of BC-derived exosomes validated the aberrant expression of exosomal miRNAs. In a recent study, miR-375-3p was notably downregulated and suppressed in BC by blocking the Wnt/β-catenin pathway and the level of the downstream molecules like cyclin D1 and c-Myc, thereby repressing BC cell growth by targeting FZD8 (Li et al., 2020). miR-204-5p is suggested to be a powerful pan-cancer suppressor, and reestablishing its levels may be a potential cancer treatment strategy (Yao et al., 2020).

#### lncRNAs

lncRNAs have been linked to human cancers and may function in carcinogenesis and cancer progression (Wei et al., 2017). The mechanism of action of lncRNAs varies depending on the circumstances; nevertheless, recent research suggests the importance of the interaction between lncRNAs and microRNAs. The exosomal lncRNA HOTAIR has been postulated to be a putative target treatment for lung cancer. It promotes proliferation of lung cells through sponging miR-203 (Zhang et al., 2020). The lncRNA UFC1, transmitted *via* exosomes, possibly binds to EZH2 to inhibit PTEN levels and stimulate the PI3K/Akt signaling pathway, hence promoting the tumorigenesis of non-small cell lung cancer (NSCLC) (Zhang et al., 2020). The exosome-delivered lncRNA ZFAS1 can promote gastric cancer (GC) progression. It indicates that ZFAS1 is a potent diagnostic and prognostic biomarker for GC (Pan et al., 2017). lncRNA FAL1 functions as an oncogenic lncRNA and enhances cancer progression by acting as a ceRNA of miR-1236 in HCC cells (Li et al., 2018).

Accumulating evidence has shown that lncRNAs could function as either an oncogenic or a tumor suppressor gene. The exosomal LBX1-AS1 has been reported as a tumor suppressor. It suppresses oral squamous cell carcinoma (OSCC) cells by invading through the miR-182-5p/FOXO3 pathway. RBPJ, a recombination signal binding protein, is frequently exploited as an activation marker of Notch signaling. The LBX1-AS1/miR-182-5p/FOXO3 pathway is stimulated and tumor growth is inhibited by macrophage-derived exosomes with overexpressed RBPJ (Ai et al., 2021). lncRNA HAND2-AS1 suppresses the progression of triple-negative breast cancer by regulating the release of MSC-derived exosomes, which have encapsulated miR-106a-5p (Xing et al., 2021). Exosomes derived from normal cells transfer PTENP1 that inhibits bladder cancer progression. It suggests that exosome-derived PTENP1 mediates normal cell-to-bladder cell communication during BC tumorigenesis (Zheng et al., 2018).

#### circRNAs

circRNAs belong to a class of covalent circular endogenous RNAs formed by the 3′ splice donor of pre-mRNA covalently linked to the 5’ splice acceptor in the reverse order. The circRNAs play a crucial role in the progression of a diverse range of cancers. They interact with miRNAs by stable complementary binding and serve as efficient miRNA sponges, thereby modulating post-transcriptional expression of downstream target genes. Moreover, circRNA could be delivered to tumor cells or normal cells by exosomes and have a regulatory role in tumor progression. Through the expression profile of HCC tissues, circMAN2B2 was shown to be highly expressed and closely related with the prognosis of HCC patients. Furthermore, circMAN2B2 sponges miR-217, which will be able to overexpress the MAPK1 signaling pathway and enhance HCC progression (Fu et al., 2021). circARHGAP10 has been shown to be elevated in both NSCLC cells and serum-derived exosomes. Exosomal transfer of circARHGAP10 promotes the proliferation of NSCLC *via* the miR-638/FAM83F axis (Fang et al., 2022). In another study, circNRIP1 has been shown to function as a sponge for miR-149-5p in order to regulate the level of AKT1 and subsequently play a tumor-promoting role in GC (Zhang et al., 2019).

Numerous circRNAs have been discovered to have tumor-suppressive properties against a number of cancers. For example, exosomal circ-0051443 has been reported to sponge miR-331-3p in order to suppress BAK1 and halt HCC progression (Chen et al., 2020). The circular RNA, namely, hsa_circ_0072309 prevents progression of GC cells by inhibiting PI3K/AKT signaling *via* activating PPARγ/PTEN signaling (Guo et al., 2022).

### Resisting cell death

Apoptosis, the programmed cell death, can be provoked by both intrinsic and non-cell autonomous signals that sense abnormality in various cell cycle processes (Hersey and Zhang, 2003). It involves the regulated deterioration of the chromosomes and other crucial cellular organelles by specialized enzymes (like caspases), the shriveling and disintegration of the cell, and its endocytosis by surrounding cells or tissue-surveilling phagocytes (Hanahan and Weinberg, 2016). Necroptosis, conceptualized as the gradual breakdown of a dying cell, could be triggered under different conditions, like oxygen and energy distress, viral infection, and inflammation (Gong et al., 2019). During necroptosis rupture, the dying cells release their contents and their remains which are left behind, which act as immunogenic debris that is able to attract (or aggravate) an immune inflammatory response (Najafov et al., 2017). The cell death program operative during autophagy functions as an organelle recycling system that helps cells cope with challenges such as nutrition destitution (White, 2015). These three distinct cell death-triggering mechanisms must be variably evaded or dampened by cancer cells in order to continue their proliferative expansion and phenotypic evolution to states of intense malignancy. Oncogenic and tumor-suppressive exosomal ncRNAs may act as both a promoter and inhibitor of these cell death mechanisms.

#### miRNAs

miR-205 might function as a proto-oncogene in ovarian cancer progression. Ovarian cancer cell SKOV3 cell-derived exosome shuttle miR-205 could attenuate the apoptosis of receptor SKOV3 cells *via* regulating VEGFA (Wang et al., 2019). Melanoma stem cells deliver their exosomal miR-4535 to melanoma parental cells (MPCs), where it amplifies metastatic colonization of MPCs by inhibiting the autophagy pathway (Liu et al., 2022). Human umbilical cord mesenchymal stem cells (hUCMSC)-derived exosomal miR-224-5p modulates breast cancer autophagy in cells by involving HOXA5 (Wang et al., 2021). Silencing of exosomal miR-25 released from cancer cells targets SIK1 and promotes the apoptotic sensitivity of liver cancer stem cells in order to promote HCC tumorigenesis (Fu et al., 2022). Exosomes released from cancer-associated fibroblasts (CAFs) loaded with miR-181d-5p could be taken up by breast cancer cells and impair apoptosis *via* downregulating CDX2 and HOXA5 (Wang et al., 2020). CAF-exosomal miR-148b-3p has been reported to reduce apoptosis in bladder cancer cells. This effect can be reverted by PTEN overexpression by downregulation of the Wnt/β-catenin pathway (Shan et al., 2021).

miR-1910-3p has a tumor-suppressive role as it could be transported *via* exosomes to mammary epithelial cells and breast cancer cells, where it results in suppression of the MTMR3 level, and activates the NF-κB and wnt/β-catenin signaling pathway, hence promoting autophagy in cancer cells (Wang et al., 2020). Ectopic expression of miR-451a is able to perturb HCC growth and tumor angiogenesis *via* apoptosis, both *in vitro* and *in vivo,* with LPIN1 being its target gene (Zhao et al., 2019).

#### lncRNAs

Exosome-encapsulated lncRNA CEBPA-AS1 could inhibit tumor apoptosis and works as a non-invasive biomarker in GC (Piao et al., 2020). Mesenchymal stromal cell (MSC)-secreted extracellular vesicles promote multiple myeloma carcinogenesis *via* lncRNA LINC00461, that has substantially enhanced levels in patients with multiple myeloma. LINC00461 enhances progression and inhibits apoptosis of multiple myeloma cell lines. It exerts its effect *via* modulating miR-15a/16 and BCL-2 (Deng et al., 2019). Knockdown of lncRNA SBF2-AS1 in exosomes produced by M2 macrophages promotes miR-122-5p expression and decreases XIAP levels, indicating lncRNA SBF2-AS1 could inhibit apoptosis by modulating XIAP *via* miR-122-5p in pancreatic cancer (Yin et al., 2020). LINC00470 plays an oncogenic role in glioblastoma multiforme (GBM)-derived exosome by binding to miR-580-3p, regulating the levels of WEE1 and activating the PI3K/AKT/mTOR pathway. Hence, it inhibits autophagy and enhances the progression of glioma cells (Ma et al., 2021).

Certain lncRNAs could promote cell death of tumor cells by inducing autophagy, apoptosis, or/and necrosis and, hence, play a tumor-suppressive role. SNHG9 is overexpressed lncRNA in papillary thyroid cancer (PTC) cell-derived exosome, where it enhances cell apoptosis, while, on the other hand, it inhibits cell autophagy of normal thyroid epithelial cell nthy-ori-3 *via* the YBOX3/P21 pathway (Wen et al., 2021). Tumor-associated macrophages (TAM)-exosomes consist of high levels of lncRNA H19, which significantly enhances autophagy in bladder cancer cells when treated with TAM-exosomes (Guo et al., 2022). In osteosarcoma, exosomal lncRNA OIP5-AS1 could promote autophagy *via* miR-153 and ATG5 (Li et al., 2021).

#### circRNAs

Exosomal circRNA_400068 exerts an oncogenic effect *via* inhibiting apoptosis and, thereby, boosting the progression of renal cell carcinoma through the miR-210-5p/SOCS1 axis (Xiao and Shi, 2020). Exosomal circ-PVT1 functions in cisplatin resistance by regulating apoptosis and autophagy through the miR-30a-5p/YAP1 axis in GC (Yao et al., 2021). Similarly, circRNA UBE2Q2 enhances the malignancy of GC through negative regulation of STAT3-mediated autophagy and glycolysis (Yang et al., 2021). Decreased circRELL1 is related with an advanced tumor node metastasis (TNM) stage and a bleak outcome while elevated circRELL1 promotes EPHB3 to suppress GC autophagy by acting as a sponge of miR-637 *in vitro* and *in vivo* (Sang et al., 2022).

### Enabling replicative immortality

Cellular senescence, which restricts the cell division number, functions as a barrier to cancer progression. This natural process, known as the Hayflick phenomenon, is associated with aging, resulting in telomere shortening (Calcinotto et al., 2019). Cancer cells are widely believed to have circumvented this brake and, hence, have unlimited replicative potential. Telomerase, which inserts telomeric repeats to the termini of telomeric DNA, is overexpressed in most of human malignancies and results in an unlimited replication potential (Loaiza and Demaria., 2016).

#### miRNAs

Human telomerase reverse transcriptase (hTERT), a c-Myc target gene, facilitates cancer cell immortality by promoting the generation of telomeric DNA. miR-185, a newly discovered pro-senescence miRNA present in human serum, when secreted *via* exosomes, targets POT1 to promote telomere dysfunction and cellular senescence. Moreover, the enhanced expression of miR-185 causes telomere dysfunction in cancer cells and primary human somatic cells (Li et al., 2020). In cervical cancer cells, telomerase is found to be linked with the regulation of radio-sensitivity by downregulating hTERT. Cervical cancer cells may be radio-sensitized by administration of exosomal miR-22*.* Overexpressing miR-22 expression *via* transfection results in the reduction of the MYCBP gene expression and consequent suppression of hTERT, and hence, enhancement of radio-sensitivity in cervical cancer cells (Konishi et al., 2020). Upon administration of exosomal miR-22 to the SKG-II cells, the expression of MYCB and hTERT is markedly reduced and is correlated with increased radio-sensitivity.

#### lncRNAs

A known lncRNA, TERRA (telomeric repeat-containing RNA) regulates replicative immortality by inhibiting telomerase. TERRA is transcribed from telomeric ends and serves as a tumor suppressor, which can negatively regulate the activity of telomerase. A cell-free form of TERRA (cfTERRA) composed of a nucleoprotein component of extracellular microvesicular exosomes in cancer cell culture and human blood plasma has been reported. These cfTERRA-harboring exosomes were found to induce inflammatory cytokines in peripheral blood mononuclear cells (PBMCs) (Wang et al., 2015).

#### circRNAs

circWHSC1, a highly expressed exosomal circular RNA in ovarian cancer, can act as a pro-tumorigenic circular RNA. It is capable of adsorbing miR-145 and miR-1182 and, thereby, upregulating the levels of downstream targets MUC1 and hTERT, enhancing cancer cell proliferation and invasion. Furthermore, peritoneal mesothelial cells serve as recipient cells and take up circWHSC1-rich exosomes (Zong et al., 2019).

### Inducing angiogenesis

Tumor cells acquire the trait to induce angiogenesis to fulfill their elevated need for nutrients and oxygen, which would otherwise be limited by the intrinsic diffusion limit of oxygen and nutrients (Aguilar-Cazares, et al., 2019). By producing new blood vessels, tumor cells not only ensure they get oxygen and nutrients but also eliminate toxic metabolic waste and initiate the hematogenous metastatic process (Zuazo-Gaztelu and Casanovas, 2018). Angiogenesis induction is a crucial step in tumor development and progression and is fueled by a variety of cancer cell-derived signaling molecules. Exosomes impart both pro- and anti-angiogenic characteristics by modulating cellular contents and acting as cancer cell disposal units (Ludwig and Whiteside, 2018).

#### miRNAs

As a member of the miR-200 family, miR-141 governs a number of biological processes in both healthy and diseased situations. It does so by binding to specific targets and controlling distinct signaling pathways, particularly in areas like angiogenesis and tumorigenesis. Tumor exosome-encapsulated miR-141 facilitates angiogenesis and malignant development of lung cancer, with its target being GAX (Wang et al., 2021). Exosome-encapsulated miR-23a inhibited PTEN, accelerating the growth of GC by increasing angiogenesis (Du et al., 2020). miR-619-5p loaded in NSCLC-derived exosomes enhances angiogenesis and malignancy by inhibiting RCAN1 (Kim et al., 2020). miR-1290 packaged in exosomes can be transferred to endothelial cells and downregulate SMEK1, which in turn, results in increased tumor angiogenesis *via* a VEGFR2-mediated action (Wang et al., 2021). miR-210, encapsulated in hepatoma cell exosomes, may be delivered to endothelial cells and induce pro-angiogenesis effects *via* targeting SMAD4 and STAT6 (Lin et al., 2018).

In contrast, certain exosomal miRNAs are known to be negatively associated with angiogenesis and exert an antitumor effect. In nasopharyngeal carcinoma (NPC) carcinogenesis, tumor exosome-associated miR-9 possesses an extracellular anti-angiogenic function. Exosomal miR-9 suppresses angiogenesis in NPC *via* targeting MDK and modulating the PDK/AKT pathway (Lu et al., 2018).

#### lncRNAs

lncRNA RAMP2-AS1 participates in the genesis and proliferation of malignant tumors. Chondrosarcoma cell-derived exosomal lncRNA RAMP2-AS1 is shown to facilitate angiogenesis through the miR-2355-5p/VEGFR2 axis (Cheng et al., 2020). The hypoxic microenvironment drives tumor cells to generate exosomes and enhance tumor angiogenesis. In the hypoxic tumor microenvironment, the expression of lncRNAs varies, and some of them can be contained in exosomes. lncRNA UCA1 is elevated in exosomes released by hypoxic pancreatic cells and can be delivered to HUVECs, boosting angiogenesis by modulating the miR-96-5p/AMOTL2/ERK1/2 axis (Guo et al., 2020). Exosome-derived FAM225A has been suggested to be a therapeutic target for esophageal squamous cell carcinoma (ESCC) patients. It upregulates the NETO2 and FOXP1 levels by acting as a sponge of miR-206 and accelerating ESCC progression and angiogenesis (Zhang et al., 2020).

#### circRNAs

Exosomal circRNA-100338 is elevated in highly malignant hepatocellular carcinoma (HCC) cells compared with low metastatic ones. It improved the metastatic capability of HCC cells and promoted angiogenesis of human umbilical vein endothelial cells (HUVECs) (Huang et al., 2020). Internalized circRNA-100338 interacts with NOVA2, an RNA-binding protein that regulates vascular formation, in HUVECs transfected with biotin-labeled circRNA-100338. The plasma levels of circ-29 in GC patients are elevated as compared to those of normal humans. The elevated circ29 acts as a competitive endogenous RNA (ceRNA) by combining with miR-29a to enhance the highly malignant phenotypes of HUVEC cells by the VEGF pathway, while downregulated circ29 is found to have the opposite effect (Li et al., 2021).

### Activating invasion and metastasis

Invasion and metastasis is a multi-stage process, involving neoplastic cell ingression into the vasculature, persistence in the circulation, subsequent invasion, and eventually colonization of remote organs, cancer cell dispersal, and stabilization in the microenvironment in order to facilitate tumor progression (Fares et al., 2020). Numerous research works have indicated that the tumor cells interact with one another, and the neighboring stromal cells may result in the development and progression of metastatic tumor. This invasion–metastasis cascade encompasses a variety of biological alterations that facilitates cancer cell penetration into healthy tissues prior to intravasation into blood and lymphatic vessels (Krakhmal et al., 2015). Exosomal ncRNAs play a critical function in the tumor microenvironment and the procedure of promoting and impeding malignant tumor metastasis (Fan et al., 2018).

#### miRNAs

miR-208a encapsulated in exosomes derived from BMSCs has been shown to foster the malignant phenotype of osteosarcoma cells. PDCD4 is the target of miR-208a, as it is elevated, and the ERK1/2 signaling pathway is suppressed after being treated with miR-208a inhibitor-loaded exosomes (Qin et al., 2020). The exosomes loaded with miRNA released from malignant oral squamous cell carcinoma (OSCC) cells promote cell growth, migration, and invasion of cancer cells. By specifically targeting DENND2D expression *via* binding to its 3′UTR, exosomal miR-1246 was showcased as a metastasis-supporting characteristic, which involves enhanced invasion in OSCC (Sakha et al., 2016). The plasma levels of exosomal miR-92a-3p are diminished post tumor resection, and its high exosomal level is strongly correlated with HCC metastasis, implying that exosomal miR-92a-3p can serve as a dynamic and effective diagnostic biomarker for HCC (Yang et al., 2020). Exosomes produced from high-metastatic HCC communicate metastatic capacity to recipient cancer cells by transmitting miR-92a-3p. Through selective suppression of the tumor suppressor gene PTEN, miR-92a-3p-activates Akt/Snail, thereby promoting EMT and carcinogenesis of HCC.

miR-3940-5p behaves as a tumor suppressor. Exosomes from mesenchymal stem cells deliver miR-3940-5p to colorectal cancer cells (CRCs), resulting in ITGA6 downregulation and TGF-β1 signaling impairment, and ultimately, the decline in invasive and metastatic potential of CRC cells and tumors (Li et al., 2021). It is found that miR-3607-3p is concentrated in the natural killer (NK) cell-derived exosomes and transferred to pancreatic cancer (PC) cells. It is demonstrated to suppress proliferation, invasion, and migration of PC cells by using IL-26 as a direct target (Sun et al., 2019).

#### lncRNAs

In consistence with various studies, it has been indicated that exosomal lncRNAs play a role in the invasion and metastasis of numerous cancers. Castration-resistant prostate cancer cell-secreted exosomes were found to be directly internalized into prostate cancer (PCa) cells, transferring HOXD-AS1 and modulating the miR-361-5p/FOXM1 axis (Jiang et al., 2021). HIF-1α elevates the PCGEM1 levels under hypoxic conditions, and it can be enveloped into exosomes, which promotes GC cell invasiveness and metastatic potential. PCGEM1 is able to maintain the stability and SNAI1 from getting degraded. SNAI1 facilitates EMT and, hence, enhances the invasion and metastatic potential of GC (Piao et al., 2021). Elevated levels of lncRNA LINC01711 in ESCC tissues are linked with poor prognosis. The progression and migration of ESCC cell lines is inhibited by silencing LINC01711. It is established as a ceRNA that represses miR-326 and upregulates the expression of fascin actin-bundling protein 1 (FSCN1) and hence improves the incidence and progression of ESCC (Xu et al., 2021).

#### circRNAs

The circular RNA hsa-circ-0004277 encourages epithelial–mesenchymal transition (EMT) in peripheral cells and a malignant phenotype in hepatocellular carcinoma. It has been demonstrated that the circ-0004277-exosome from HCC cells increases circ-0004277 expression in HL-7702 cells, induces invasiveness, and boosts the EMT process (Zhu et al., 2021). Hypoxia-derived exosomal circ-133 in CRC is delivered into normoxic cancer cells and enhances cell migration *via* the miR-133a/GEF-H1/RhoA axis (Yang et al., 2020). Exosomal circ007293 can be transported to papillary thyroid carcinoma (PTC) cells and participate in altering PTC cell malignant phenotypes. Exosomal circ007293 inhibits miR-653-5p activity *via* acting as a sponge for miR-653-5p, and hence, enhancing PAX6 levels in PTC cells and increasing tumor cell metastasis and EMT (Lin et al., 2021). Exosomal circ-PDE8A stimulates tumor invasion by miR-338/MACC1/MET/AKT or ERK pathways. Circulating tumor-secreted circ-PDE8A can be secreted into the bloodstream *via* exosome transfer, and plasma exosomal circ-PDE8A is associated with tumor invasion and prognosis in patients with pancreatic ductal adenocarcinoma (PDAC) (Li et al., 2018).

### Reprogramming of energy metabolism

Cancer cells routinely modify their metabolism in order to generate adenosine triphosphate (ATP) promptly for boosting macromolecular synthesis and maintaining an optimum homeostatic redox balance (Martinez-Outschoorn et al., 2017). Unlike normal cells, tumor cells display different metabolic characteristics, involving excessive glucose uptake, a greater reliance on aerobic glycolysis, elevated glutamine uptake and glutaminolysis, and altered lipid metabolism (Vander Heiden and DeBerardinis, 2015). The primary objective of metabolic reprogramming in cancer cells is to maintain balanced energy expenditure and enable biomass production in order to facilitate cancer cell proliferation (Pavlova and Thompson, 2016).

#### miRNAs

Exosomal miR-105 is stimulated by the oncoprotein MYC in cancer cells and promotes MYC signaling in CAFs to drive a metabolic program. It enables CAFs to demonstrate varied metabolic characteristics in response to alterations in the metabolic environment. In ample availability of nutrients, miR-105-reprogrammed CAFs increase glucose and glutamine metabolism to fuel neighboring cancer cells. Upon encountering a decrease in nutrient levels and the build-up of metabolic byproducts, the CAFs aid in conversion of lactic acid and ammonium into energy-rich metabolites to detoxify metabolic wastes. Thus, miR-105-directed metabolic reprogramming of stromal cells promotes tumor growth by controlling the metabolic environment (Yan et al., 2018). Researchers explored whether melanoma-derived exosomes could alter normal human adult dermal fibroblast (HADF) metabolism, hence adding to optimal pre-metastatic niche conditions. Their observation of enhanced glycolysis and diminished OXPHOS in normal HADF in contact with human melanoma-derived exosomes (HMEX) and enhancement of the “Warburg effect” is in consistence with results regarding the capacity of tumor exosomes to reprogram stromal cells. They demonstrated that HMEX and specifically its microRNAs miR-155 and miR-210 are able to remodel the metabolism of stromal fibroblasts in order to promote aerobic glycolysis (Shu et al., 2018). Pyruvate dehydrogenase E1 subunit alpha 1 (PDHA1) is reduced dramatically in cisplatin (DDP)-resistant SKOV3 and DDP-resistant ovarian tumor tissues, whereas miR-21-5p is considerably enhanced as compared to controls. Moreover, miR-21-5p is highly elevated in SKOV3/DDP exosomes relative to SKOV3 exosomes. It has been indicated in a study that SKOV3/DDP exosome therapy reduced the cisplatin sensitivity of SKOV3 cells and increased cell survival and glycolysis through PDHA1 inhibition *via* exosomal miR-21-5p. This miRNA from DDP-resistant SKOV3 OC cells was reported to induce glycolysis and suppress chemosensitivity of its progenitor SKOV3 cells *via* targeting PDHA1 (Zhuang et al., 2021).

#### lncRNAs

Malignant cells and CAFs established a network of interactions inside the microenvironment of a tumor. The findings established by a group of researchers suggest a novel metabolic modulatory role of CAF–exosomal lncRNA in breast malignancies by demonstrating that the SNHG3/miR-330 signaling axis altered the metabolism and proliferation of breast tumor cells by altering PKM at the post-transcriptional level (Li et al., 2020).

#### circRNAs

Generally, metastatic neoplasms, like colorectal cancer (CRC), depend on ATP synthesis *via* aerobic glycolysis for accelerated growth. From a panel of dysregulated circRNAs, ciRS-122 has been projected to sponge miR-122 in drug resistance-resistant CRC cells. Furthermore, the ciRS122 level in serum exosomes has been verified to be positively linked with chemoresistance. Exosomes could deliver ciRS-122 from drug-resistant cells to drug-sensitive cells, where glycolysis and drug resistance are augmented by inhibiting miR-122 and upregulating PKM2. Furthermore, the suppression of ciRS-122 significantly decreases glycolysis and reverses oxaliplatin resistance in CRC (Wang et al., 2020).

circ_0094343 is considerably downregulated in CRC and when transported by exosomes, it plays a suppressive function against the aggressiveness of HCT116 cells. It sponges miR-766-5p, which targets and regulates TRIM67. Moreover, mechanistic validation indicated that circ_0094343 can repress HCT116 cell proliferation, clone formation, glycolysis, and chemotherapy resistance through the miR-766-5p/TRIM67 axis (Li and Li, 2022).

### Evading immune destruction and promoting tumor inflammation

Due to its ability to evade immune detection and generate an immunosuppressive environment, cancer can hinder attempts to mount a robust antitumor response. Immune escape, according to the immune-editing notion, is essential for tumor survival (Vinay et al., 2015). Tumor immune escape (TIE) mechanisms include abnormalities in tumor antigen presentation that allow tumors to avoid immune system identification, perturbations in the tumor death pathway to enhance resistance to cytotoxic immune responses and metabolic aberrations to promote tumor evasion, and establishment of stem cell-like phenotypes in order to avoid immune-based detection and elimination (Shimizu et al., 2018). Moreover, TIE is influenced by various cytokines in the tumor microenvironment (TME), aberrant expression of immunological checkpoint molecules on tumor or immune cell surfaces, and certain immunosuppressive cells. These characteristics may combine to facilitate TIE, causing a low rate of response to immunotherapy in many cancers (Muenst et al., 2016). Exosomal ncRNAs involved in TIE are currently emerging as attractive prospective targets for anticancer treatment. Several investigations have found that exosomal ncRNAs play a crucial role in TIE (Chen et al., 2019).

#### miRNAs

Despite their role as a barrier to the effector arm of the antitumor immune response, the immunosuppressive mechanism of lymphatic endothelial cells during tumorigenesis within the microenvironment is poorly defined. The intercellular crosstalk within the TME has been attributed to exosome-derived miRNAs. A decrease in CD8^+^ T cell immunity by activation of JAK2/STAT3 signaling is triggered by the exosomal microRNA miR-1468-5p, released by cervical cancer cells. The microRNA augmented the PD-L1 expression and vascularization within the lymphatic system by suppressing HMBOX1-SOCS1 expression. The findings lend credence to a mechanism for the growth of tumors dependent on lymphatic immunosuppression (Zhou et al., 2021). Similarly, miR-1290 encapsulated within the GC cell-derived extracellular vesicle and lowered the proliferation of T cells by modulation of the Grhl2/Zeb1/PD-1 axis, facilitating the immune evasion (Liang et al., 2021).

MSCs are capable of suppressing the immune system and aiding tumor cells in evading immunological responses. An interaction strategy between colorectal cancer cells and MSC-EVs has been presented, in which miR-222 originating from MSC-EVs commits the post-transcriptional regulation on ATF3, which, therefore, activates the AKT pathway and encourages the tumorigenesis of CRC and immune evasion (Li S. et al., 2021). Gastric cancer (GC) extracellular vesicle (EV) encapsulated miR-675-3p aid in the immune evasion of GC cells by repression of CXXC4 and boosting the expression of PD-L1 *via* the MAPK signaling pathway. The favorable cytokine profile in the TME triggers the rapid amplification of activated cytotoxic NK cells, which is perceived as an important prognostic indication (Li et al., 2020). Moreover, miR-21 conveyed by BMDM exosomes accelerates glioma cell growth and inhibits apoptosis by limiting PEG3 (paternally expressed gene 3). This further facilitates immune escape of glioma cells by increasing the tumor burden and expression of PCNA and Ki67, prominent nuclear markers to demonstrate proliferative phase of the cell cycle, and decreasing the CD8^+^ T cell population in glioma. Depleting miR-21 or reintroducing PEG3 reinstated the proliferative capacity of CD8^+^ T cells and boosted the cell cytotoxicity and IFN-γ levels, while decreasing the activity of cancer cells and the level of TGF-β1, as demonstrated by Yang et al. (2020).

Several miRNAs with tumor-suppressing ability act to regulate the immune suppressive trait of cancer. Adipose-derived mesenchymal stem cells (adMSCs) have immunomodulatory property and the ability of triggering *de novo* regulatory T cells. Exosomes derived by adMSCs encapsulating miR-15a are taken by CRC cells, resulting in a decline of the KDM4B and HOXC4 levels, which in turn reduces the production of PD-L1 that prevents CRC cells from immune evasion. Additionally, this cascade of actions also inhibits CRC cell malignancy by stifling their proliferation, invasion, and metastasis (Liu et al., 2021). Based on the findings, tumor suppressor miR-186 entrapped in NK cell-derived exosomes has diminished levels in high-risk neuroblastoma. The longevity and motility of MYCN-amplified neuroblastoma cells are impaired by ectopic delivery of miR-186 to NK cells and neuroblastoma cells, and TGF-dependent suppression of NK cytotoxicity is averted. Irrespective of the activation status of NK cells, the exosomes generated by them are capable of eliminating MYCN-amplified neuroblastoma cell proficiently, apparently suggesting that the miR-186 level is accountable for the cytotoxic effect, and NK exosomes are resilient to TGF-β1-dependent suppression (Neviani et al., 2019).

#### lncRNAs

γδT cells act as a prominent constituent of tumor-infiltrating lymphocytes (TILs) in breast cancer. The subpopulation CD73+γδT1 cells remain the major regulatory T cells (Tregs) in breast cancer. The expression of SMAD5 in γδT1 cells gets upregulated *via* transfer of exosomal lncRNA SNHG16 that serves as a ceRNA by acting as a sponge of miR-16–5p and, hence, potentiates the TGF-β1/SMAD5 pathway to enhance CD73 levels (Ni et al., 2020).

NK cells are an innate part of the immune system and are in command of eradicating cancer cells either directly or by sequestering cytokines upon activation. In malignancies, like ESCC, NK cell functionality is repressed or dysfunctional, leading to immune escape (Kim., 2007). Exosomes released by metastatic CRC cells have a proven role in immunologically dampening NK cells, as well as a strategy to accomplish this goal. The consequences of exosomes on NK cells have been determined by tracking their proliferative ability, cytotoxic capacity, secretion of interferons (IFN-γ), and perforin and granzyme B expression levels. Employing next-generation sequencing, the vital lncRNAs within exosomes and the genes they influence have been traced out. Secreted exosomes by CRC cells have indeed been demonstrated to transmit the lncRNA SNHG10 that impairs NK cell activity and enhances tumor growth. To stimulate the TGF-signaling pathway, it facilitated the production of inhibin subunit beta C (INHBC), which in response suppressed NK cytotoxicity (Huang et al., 2021).

Numerous studies have shown PD-1 as the predominant inhibitory receptor in tumor immunology. Exosomal participation in the KCNQ1OT1/miR-30a-5p/USP22 axis-mediated control of PD-L1 provides a deeper understanding of immune escape of CRC. The expression of lncRNA KCNQ1OT1 was found to be markedly increased simultaneously in both, exosomes generated from tumor cells and tumor tissues. The lncRNA KCNQ1OT1 supports colorectal tumorigenesis by modulating PD-L1 ubiquitination and limiting CD8^+^ T-cell response *via* the autocrine effect of CRC exosomes (Xian et al., 2021). The prominent lncRNA TUC339, overexpressed in exosomes derived of HCC cells, promotes HCC cellular proliferation and obstructs cell adherence with an extracellular matrix on transmission to adjacent tumor niche *via* exosomes. On the basis of recent evidences, the transfer of lncRNA to the immune cells like macrophages has been promulgated *via* exosomes, leading to alteration in their phenotype. For example, in macrophages, lncRNA TUC339 modulates cytokine secretion, phagocytic activity, and polarization toward the M1/M2 state (Li et al., 2018). In a recent study, exosomes secreted by renal cell carcinoma (RCC) encases lncARSR, which leads macrophages to polarize from M1 to M2, to secrete cytokines, engage in phagocytosis, and initiate angiogenesis, hence substantially contributing in the development of malignancies. Additionally, by serving as competing endogenous RNA for miR-34/miR-449-5p, lncARSR encourages polarization of macrophages by activating the STAT3 pathway (Zhang et al., 2022).

#### circRNAs

PD1 is a negative costimulatory receptor that is important for suppressing T-cell activation and is associated with SHP2. In addition, SHP2 plays an imperative role in oncogenic KRAS-driven malignancies, promoting tumor development. Enhanced circUSP7 levels blunt the clinical efficiency of anti-PD-1 therapy orchestrated *via* the exosomal circUSP7/miR-934/SHP2 axis. In NSCLC patients, circUSP7 promotes tumor progression and is critical for immune evasion (Chen et al., 2021). Similarly, circGSE1 facilitates immunological escape of HCC by facilitating the proliferative ability of Tregs *via* modulating the miR-324p/TGFBR1/Smad3 axis (Huang et al., 2022). Correspondingly, in patients with HCC, enhanced levels of circUHRF1 imply NK cell malfunction and a poor clinical outlook. CircUHRF1 restricts NK cell-derived IFN-γ and TNF-α secretion and is predominantly secreted in plasma exosomes of HCC patients. Elevated levels are linked to lower the NK cell percentage and tumor infiltration. Furthermore, circUHRF1 inhibits NK cell function by elevating TIM-3 levels by the inhibition of miR-449c-5p (Zhang et al., 2020).

By serving as a miR-141-3p sponge, exosomal hsa-circ-0085361 (circTRPS1) has been associated with metastatic spread of bladder cancer cells. GLS1-mediated glutamine metabolism was revealed to be implicated in circTRPS1-mediated perturbations *via* integrated metabo-transcriptomics study. Exosomal-circTRPS1 secreted by knocked-down breast cancer cells hindered the exhaustion of CD8^+^ T lymphocytes and impeded breast cancer cell’s propensity to become malignant. Therefore, it might be concluded that the circTRPS1/miR-141-3p/GLS1 axis regulates the equilibrium of intracellular reactive oxygen species (ROS) generation and exhaustion of CD8^+^ T cell *via* breast cancer exosomes (Yang et al., 2022). It has also been suggested that exosome-encapsulated circ_6790 released from MSC downregulates S100A11 in PDAC cells and, thereby aids in immune evasion. Along with the antitumor effects of circ_6790-loaded exosomes derived from BM-MSC, their supporting role in enhancing the killing effects of activated T cells has been demonstrated. Such exosomes diminished the levels of PD-L1 and CTLA-4 in PDAC cells co-cultured with exosomes and T cells in addition to reducing the secretion of IFN-γ and TNF-α (Gao et al., 2022).

## Conclusion

Initially, cancer hallmarks were defined as the attainment of functional abilities that enable cancer cells to survive, proliferate, and metastasize. Later on, it has been found that exosomes facilitate information exchange among cells facilitating tumor cell development and progression. Recent years have seen a surge in studies focusing on exosomal ncRNAs, revealing important functions of these molecules in the progression of cancer and suggesting potential new uses for them. Among the several ncRNAs, miRNAs, lncRNAs, and circRNAs are considered the mainstream regulatory molecules. Exosomal ncRNAs play a role in oncogenic spread, immunological regulation, and the establishment of pre-metastatic niches.

In this review, we have called attention to the biological attributes of exosomes and showcased an extensive update about the roles of exosomal ncRNAs in tumor hallmarks, especially growth, metastasis, angiogenesis, replicative immortality, cell death, metabolic regulation, and immune modulation. The exosomal ncRNA interacts with the promoter or enhancer region and modulates the gene expression. The released ncRNAs may act as a tumor promoter in one cancer and as a tumor suppressor in another sort of cancer. This finding highlights the possibility that the roles and expression patterns of at least certain exosomal ncRNAs in cancer development and advancement are context-dependent. Concentrating on their roles as tumor suppressor and tumor promoter genes, here, we examine the functional relationship between exo-ncRNAs implicated in cancer development and progression.

It should be noted that a range of exosomal constituents can be exploited as a biomarker (diagnosis and prognosis) and treatment target of cancer. Several exosomes encapsulated ncRNAs can serve as predictive markers of associated cancers. Endogenous ncRNAs contained within circulating exosomes may also serve as a source of valuable information and can be targeted by a specialized treatment protocol. It may help in designing specific drugs and other specific inhibitors that are closely related to these RNAs, aiding in advancement toward personalized treatment regimens.

In addition to breakthroughs in mechanistic research, a key difficulty in the clinical setting that needs attention is the limitation of potentially harmful RNAs and the optimization of medication doses for exosomal therapy. Another uncharted concern in the sector is the quest to guarantee the quality and safety of new methodology applied for the isolation and utilization of exosomes. As we gain a greater understanding of the nature of exosomes, diagnostic and therapeutic tools are also advancing. Future research will most likely focus on *in vivo* models and clinical applications to help resolve these challenges. Exploratory research in this emerging segment is anticipated to provide knowledge that is highly clinically relevant and has the capability to positively transform the lives of cancer patients.
